# Supplementary figures and images for: Oldest skeleton of a fossil flying squirrel casts new light on the phylogeny of the group
Source: eLife. 2018 Oct 9;7:e39270. doi: 10.7554/eLife.39270 (PMC6177260; doi:10.7554/eLife.39270)

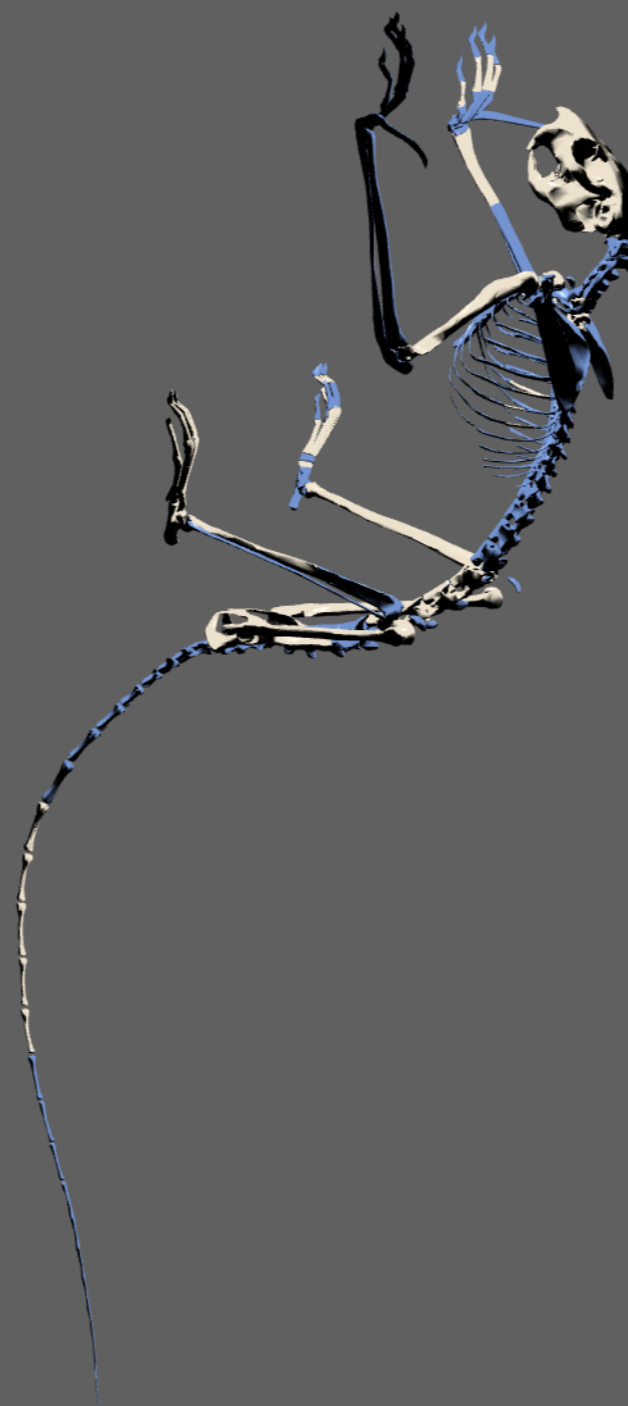

Supplement: Supplementary file 1. [file elife-39270-supp1.pdf]
