# Supplementary material for: Oldest skeleton of a fossil flying squirrel casts new light on the phylogeny of the group
Source: eLife. 2018 Oct 9;7:e39270. doi: 10.7554/eLife.39270 (PMC6177260; doi:10.7554/eLife.39270)
Supplement: Transparent reporting form [file elife-39270-transrepform.docx]

**Answers to questions for *eLife* digest**

Dear editors, we tried to keep within the suggested word limit for the digest, but this work explored many different topics, from the description of a new fossil to the evolution of the whole flying squirrel group. Furthermore, we include a note detailing the story of the finding, since the skeleton was initially mistaken for a small-sized primate.

1. **What background information would someone who is completely unfamiliar with your field need to know to understand the findings in your paper? (suggested word limit: 150 words)**

Several groups of mammals have independently evolved gliding locomotion, examples including marsupial sugar gliders, colugos and a few rodent families. However, flying squirrels are the only ones to have achieved a remarkable success, with 52 species and occurring in all northern continents. Yet their evolutionary history is not well known to the point that estimates of their time of origin – as derived from molecular and paleontological data – blatantly disagree. The oldest fossils tentatively attributed to this group are more than 36 million years old, whereas most molecular studies conclude that flying squirrels split from tree squirrels about 23 million years ago. The problem is that their fossil record is very incomplete and isolated cheek teeth is all that we know of most extinct ‘flying’ squirrels. Unfortunately, recent studies have shown that the features of the cheek teeth used to classify these fossils as flying squirrels also occur in tree squirrels. Only the adaptations of the skeleton related to gliding could unambiguously identify a fossil as a flying squirrel.

2. **What exact research question did you set out to answer and why? (suggested word limit: 75 words)**

The only way to tell if an extinct ‘flying’ squirrel truly belonged to this group is finding more complete remains that comprise the parts of the skeleton related to gliding. In turn, if we can identify an extinct species as a flying squirrel, this new evidence can be used to correct the calculations and estimate a new time of origin that could reconciliate previous paleontological and molecular results.

3. **What are the most important findings of your paper? (suggested word limit: 100 words)**

We describe the oldest, 11.6 million-year-old, skeleton of a fossil flying squirrel, *Miopetaurista neogrivensis*, from Catalonia (Spain). Even though this is a very complete skeleton, the most important finding are the diminutive wrist bones. Unlike all other gliding mammals, flying squirrels support the gliding skin membrane (patagium) thanks to a long cartilage rod attached to the wrist bones, which are unique and further differ in large- and small-sized flying squirrels. The wrist bones of *Miopetaurista* not only evidence that this was a flying squirrel but also that it belonged to the large-sized group. Considering this new evidence, we carried out evolutionary analyses combining molecular and paleontological data. The results provide older estimates for their origin, most likely around 31 to 25 million years ago, however, older dates are also possible, so we could not exclude that earlier fossils might be flying squirrels. In addition, our analyses show that *Miopetaurista* is closely related to the extant giant flying squirrel (*Petaurista*). Indeed, their skeletons are so similar that these squirrels could be considered ‘living fossils’, having changed very little in almost 12 million years.

4. **Who might eventually benefit from the findings of your study, and what would need to be done before we could achieve these benefits? (suggested word limit: 75 words)**

Molecular and paleontological data are often at odds. Here we not only describe an exceptional fossil but also show how both disciplines can be combined to provide better evolutionary trees. However, fossils are still our only direct testimony of the evolutionary process so finding new specimens can solve long-standing controversies. Therefore, the next challenge will be to find skeleton material of older flying squirrels and, if possible, of transitional forms between tree and flying squirrels.

**Additional information on the finding of the specimen:**

The story related to the finding of this skeleton is curious. It comes from a fossiliferous area including more than 200 sites that was discovered in 2002 during the construction of a huge rubbish dump near Barcelona. These sites have provided several primate specimens, including great apes as well as a smaller-sized group called pliopithecids. When the *Miopetaurista* skeleton was found only the two femora, part of the pelvis and the tail vertebrae were visible [we have pictures of that]. Considering its size (very large for a squirrel) it was initially mistaken for a pliopithecid. Once preparation began and the pelvis was exposed paleoprimatologists were disappointed to find that it was rodent, but they had found the only skeleton of an extinct flying squirrel in the world!

The diagnostic wrist bones just measure a few millimeters. They were found during the preparation of the specimen after the paleontologists insisted that all the sediment attached to the skeleton had to be screen-washed.
